# Very early recurrence predicts long-term outcome in patients after atrial fibrillation catheter ablation: a prospective study

**DOI:** 10.1186/s12872-017-0533-2

**Published:** 2017-05-08

**Authors:** Yangjing Xue, Xiaoning Wang, Saroj Thapa, Luping Wang, Jiaoni Wang, Zhiqiang Xu, Shaoze Wu, Luyuan Tao, Guoqiang Wang, Lu Qian, Lianming Liao, Baohua Liu, Kangting Ji

**Affiliations:** 1 Department of Cardiology, the Second Affiliated Hospital and Yuying Children’s Hospital of Wenzhou Medical University, Xueyuanxi Road, No 109, Wenzhou, Zhejiang 325000 China; 2 Department of Rehabilitation, the Second Affiliated Hospital and Yuying Children’s Hospital of Wenzhou Medical University, Xueyuanxi Road, No 109, Wenzhou, Zhejiang 325000 China; 3 Department of Intensive Care Unit, Zhengzhou Central Hospital Affiliated to Zhengzhou University, Tongbaibei Road, No 195, Zhengzhou, Henan 450000 China; 4 Department of Endocrinology, the Fourth Affiliated Hospital, Zhejiang University School of Medicine, Shangcheng Road, No N1, Yiwu, Zhejiang 322000 China; 5 Department of Oncology, Academy of Integrative Medicine, Fujian University of Traditional Chinese Medicine, Huatuo Road, No 1, Fuzhou, Fujian 350122 China

**Keywords:** Atrial fibrillation, Catheter ablation, Very early recurrence, Long-term recurrence

## Abstract

**Background:**

Long-term recurrence (LR) is a tendency that re-occurs within 3 months after catheter ablation for atrial fibrillation (AF). Whether very early recurrence (VER) within 7 days of post ablation is a prognostic factor of LR or not is unclear. For this reason, present study sought to examine the relationship between VER and LR.

**Methods:**

In this prospective analysis 378 consecutive patients underwent an initial catheter ablation for paroxysmal or persistent AF. The association between VER and LR was analyzed by univariate and multivariate Cox regression, as well as time-dependent receiver operator characteristic (ROC) analysis.

**Results:**

After a mean follow-up of 14.71 ± 8.58 months, 81 (65.90%) patients with VER experienced LR and were associated with lower event of free survival from LR (Log rank test, *P* < 0.001). Multivariate Cox regression analysis revealed that VER (HR = 7.02, 95% CI = 4.78–10.31; *P* < 0.001), left atrial enlargement (HR = 2.92, 95% CI = 1.88–4.54; *P* < 0.001), tendency in advanced age (HR = 1.50, 95% CI = 0.99–2.28; *P* = 0.054), and tendency in male (HR = 0.71, 95% CI = 0.50–1.01; *P* = 0.060) were independent predictors of LR. According to time-dependent ROC analysis, it was found that VER was more sensitive than common risk factors in predicting LR (0.74 vs 0.66, *P* < 0.001) and combination model further improved the C statistic for predicting LR (0.82 vs 0.66, *P* < 0.001).

**Conclusions:**

After a single procedure of catheter ablation, patients with VER were strongly associated with LR and combination of VER and common risk factors could further improve prediction of patients who were at high risk for LR.

## Background

Catheter ablation is the mainstay therapy for atrial fibrillation (AF), but the high rate of long-term recurrence (LR) is a limitation of the procedure. Non-paroxysmal AF, sleep apnea, obesity, left atrial enlargement, advanced age, hypertension, left atrial fibrosis and recurrence of AF within the first 3 months after catheter ablation have been identified to be the LR predictors [1–14]. Among them, recurrence of AF within the first 3 months is considered to be the most important predictor of long-term treatment failure [5–14]. Based on these studies, a so-called blanking period, the duration ranging from the first 7 days to 3 months post ablation, is proposed [5–15].

In the clinical practice, it has been found that lots of patients had episodes of AF as early as 7 days of post ablation. In the present study, it was aimed to examine the relationship of recurrence within 7 days, which we defined as very early recurrence (VER), and LR after 3 months. We hypothesized that VER was a prognostic factor of LR after 3 months.

## Methods

This prospective study included 378 consecutive patients with paroxysmal (*n* = 168) or persistent (*n* = 210) AF who underwent an initial ablation at the Second Affiliated hospital and Yuying Children’s Hospital of Wenzhou Medical University, from January 2013 and December 2014.

Paroxysmal AF is defined as AF that terminates spontaneously or under anti-arrhythmic drugs (AADs) within 7 days of onset. Persistent AF is defined as continuous AF sustaining for more than 7 days. Patients were excluded if they aged <20, had pregnancy, prior cardiac surgery, implanted pacemaker, chronic renal failure requiring hemodialysis, and severe mitral valve disease. All patients gave written informed consent and the study protocol was approved by our institutional review board.

For every patient, step-wise ablation strategy was performed, including circumferential pulmonary vein isolation (PVI), complex fractionated atrial electrograms, and linear ablation. The electrophysiological evaluation of PVI was bi-directional conduction block between left atria (LA) and pulmonary veins (PVs). Whether to perform additional ablation including tricuspid valve isthmus ablation, continuous fractionated atrial electrogram ablation, and LA linear ablation was decided by the operator and/or the attending physician. The ablation procedure followed the method described by Liu X et al. [16, 17].

After the ablation procedure, patients remained hospitalized under continuous electrocardiography monitoring for at least 7 days. Patients received 24 h Holter monitoring at 3, 6 and 12 months follow-ups after procedure and every 12 months thereafter. Among follow-ups, all patients were encouraged to visit doctors for ECGs or Holter monitoring for any symptoms suggestive of AT onset.

AADs continued for 1–3 months after the ablation procedure. LR was defined as any asymptomatic or symptomatic atrial tachyarrhythmia (AT) lasting >30s off AADs after the initial 3-month blanking period. VER was defined as sustained AT (lasting >30s) on or off AADs recurred within 7 days post ablation.

## Statistical analysis

Depending on the distribution, the continuous data were presented as median (25th–75th percentiles) or as mean ± SD. Categorical data were presented as counts or proportions. The differences between groups were assessed with the χ^2^ test or Fisher’s exact test for categorical data and the nonparametric Wilcoxon rank-sum test or Student test for continuous data.

Factors associated with recurrence arrhythmia during follow-ups were assessed in univariate and multivariable Cox proportional hazard models. Factors with *P* values <0.1 in univariate analyses were included in stepwise multivariate Cox regression models. Time-dependent receiver operator characteristic (ROC) curve analysis was generated to test the predictive discrimination of patients with or without LR. A two-tailed value of *P* < 0.05 was considered to indicate the statistical significance.

## Results

Baseline characteristics of patients are summarized in Table 1. AF was paroxysmal in 168 (44.44%) patients and persistent in 210 (55.56%). Only 6 patients had moderate valvular heart disease. Risk of thromboembolic (CHADS2 and CHADS-VASc Score) and bleeding (HAS-BLED Score) complications were both significantly high in patients with LR. Warfarin usage at hospital discharge tended to be more frequent in patients with LR (*P* = 0.089). Advanced age (age ≥ 65 years), female gender, increased BMI, persistent AF, hypertension, diabetes, history of heart failure (HF), decreased left ventricular ejection fraction (EF), left atrial enlargement (left atrial ≥50 mm), statins usage, and ACEI/ARB usage were significantly more frequent in patients with LR.

**Table 1:** Baseline characteristics of the Patients^a^

Variables	Total	Long-term recurrence		*P*-value
	*N* = 378	Without *N* = 255	With *N* = 123	
Age, years	65.37 ± 10.44	63.69 ± 10.40	68.85 ± 9.68	<0.001
Age ≥ 65 years, n (%)	222 (58.70%)	131 (51.40%)	91 (74.00%)	<0.001
Male, n (%)	215 (56.90%)	156 (61.20%)	59 (48.00%)	0.015
BMI, kg/m^2^	24.43 ± 3.08	24.07 ± 3.00	25.17 ± 3.15	0.001
Type of AF				
Paroxysmal, n (%)	168 (44.40%)	128 (50.20%)	40 (32.50%)	0.001
Persistent, n (%)	210 (55.60%)	127 (49.80%)	87 (67.50%)	0.001
Duration of AF, months	32.11 ± 44.82	33.44 ± 48.54	29.37 ± 35.91	0.409
Hypertension, n (%)	223 (59.00%)	141 (55.30%)	82 (66.70%)	0.035
Systolic BP, mmHg	135.17 ± 20.83	134.39 ± 21.27	136.80 ± 19.88	0.291
Diastolic BP, mmHg	82.94 ± 51.28	81.76 ± 42.34	85.39 ± 66.21	0.520
Diabetes, n (%)	56 (14.80%)	30 (11.80%)	26 (21.10%)	0.016
FBG, mmol/L	5.14 ± 1.15	5.09 ± 1.02	5.26 ± 1.39	0.117
History of HF, n (%)	46 (12.20%)	20 (7.80%)	26 (21.10%)	<0.001
Left ventricular EF, %	63.48 ± 7.52	63.82 ± 7.35	62.78 ± 7.85	0.211
Left atrial dimension, mm	40.82 ± 6.41	39.50 ± 5.72	43.56 ± 6.91	<0.001
Left atrial ≥50 mm, n (%)	39 (10.30%)	13 (5.10%)	26 (21.10%)	<0.001
Moderate valvular heart disease, n (%)	6 (1.60%)	3 (1.20%)	3 (2.40%)	0.357
CAD, n (%)	26 (6.90%)	12 (4.70%)	14 (11.40%)	0.016
Prior Stroke/TIA, n (%)	49 (13.00%)	31 (12.20%)	18 (14.60%)	0.502
CHADS2 Score	1.34 ± 1.19	1.17 ± 1.10	1.71 ± 1.30	<0.001
CHA2DS2-VASc Score	2.81 ± 1.81	2.49 ± 1.70	3.48 ± 1.83	<0.001
HAS-BLED Score	2.47 ± 1.06	2.32 ± 1.02	2.79 ± 1.07	<0.001
CRP within 24 h post-procedure, mg/dL	6.36 ± 10.27	6.38 ± 10.87	6.32 ± 8.94	0.954
Medication at hospital discharge				
Oral anticoagulant				
Warfarin, n (%)	297 (78.57%)	194 (76.10%)	103 (83.70%)	0.089
Dabigatran, n (%)	73 (19.31%)	54 (21.20%)	19 (15.40%)	0.186
Xa inhibitor, n (%)	8 (2.12%)	7 (2.70%)	1 (0.80%)	0.221
Statins, n (%)	269 (71.20%)	174 (68.20%)	95 (77.20%)	0.070
ACEI/ARB, n (%)	169 (44.70%)	103 (40.40%)	66 (53.70%)	0.015
Beta-blockers, n (%)	111 (29.40%)	70 (27.50%)	41 (33.30%)	0.239
Vaughan Williams class I or III AAD, n (%)	342 (90.50%)	235 (92.20%)	107 (87.00%)	0.109
Amiodarone, n (%)	328 (86.80%)	228 (89.40%)	100 (81.30%)	0.029
Propafenon, n (%)	14 (3.70%)	7 (2.70%)	7 (5.70%)	0.155

*BMI* body mass index, *AF* atrial fibrillation, *FBG* fasting blood glucose, *HF* heart failure, *EF* ejection fraction, *CAD* coronary artery disease, *TIA* transient ischemic attack, *CRP* C-reactive protein, *AAD* anti-arrhythmia drug

^a^Plus-minus values are means ± SD. Percentages do not sum to 100 because of rounding

**Fig. 1:**
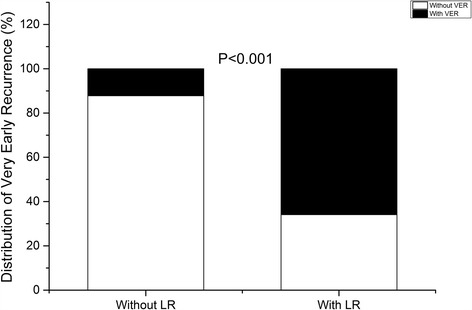
Relationship between very early recurrence (VER) and long-term recurrence (LR). In patients without LR, the constitution of VER was of 12.20%; In patients with LR, the constitution of VER was of 65.90%

After a single ablation procedure, 112 patients (29.63%) experienced VER within the first 7 days post ablation while LR cumulatively occurred in 123 (32.54%) patients after the initial 3-month blanking period. Among these 112 patients with VER, 81 (65.90%) patients experienced LR (Fig. 1).

**Fig. 2:**
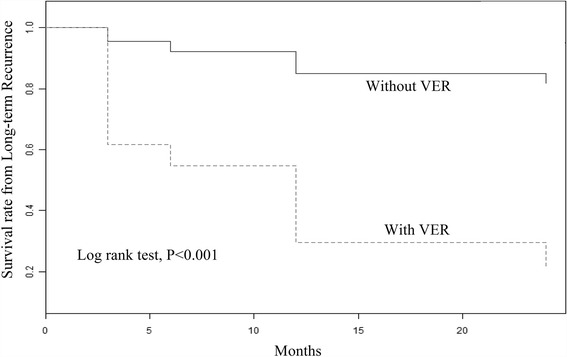
Event-free survival from the long-term recurrence (LR) for patients with and without very early recurrence (VER)

Figure 2 shows the event-free survival from the LR for patients with and without VER within 7 days. After a mean follow-up of 14.71 ± 8.58 months, patients with VER were associated with LR (Log rank test, *P* < 0.001).

Univariate Cox analysis was performed and identified that VER was associated with LR (*P* < 0.10), and similarly to the factors including advanced age (age ≥ 65 years), BMI, persistent AF, duration of AF, hypertension, diabetes, history of heart failure, left ventricular EF, left atrial enlargement, ACEI/ARB usage. In multivariable Cox regression analysis, independent predictors of LR in this study were VER (HR = 7.02, 95% CI = 4.78–10.31; *P* < 0.001), left atrial enlargement (HR = 2.92, 95% CI = 1.88–4.54; *P* < 0.001), tendency in advanced age (age ≥ 65 years) (HR = 1.50, 95% CI = 0.99–2.28; *P* = 0.054), and tendency in male (HR = 0.71, 95% CI = 0.50–1.01; *P* = 0.060) (Table 2).

**Table 2:** The results of the multivariable Cox regression analysis of the independent correlates for the LR

Parameters	OR	95% CI Low	95% CI Upp	*P*-value
VER	7.02	4.78	10.31	<0.001
Left atrial enlargement	2.92	1.88	4.54	<0.001
Advanced age	1.50	0.99	2.28	0.054
Male	0.71	0.50	1.01	0.060

*LR* Long-term recurrence, *VER* Very Early Recurrence

**Fig. 3:**
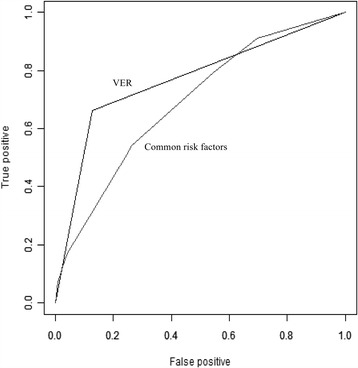
Time-dependent ROC analysis based on very early recurrence (VER) and established common risk factors (0.74 vs 0.66, *P* < 0.001), respectively

**Fig. 4:**
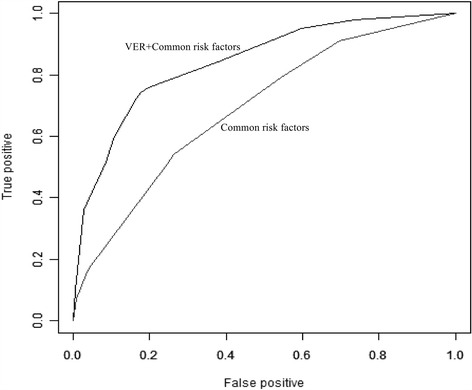
Time-dependent ROC analysis based on combined model and established common risk factors (0.82 vs 0.66, *P* < 0.001)

To further assess the potential prognostic value of VER in predicting cumulative LR, we performed time-dependent ROC analysis. C statistic for VER was significantly greater than model based on established common risk factors (left atrial enlargement, age ≥ 65, male) in this study (0.74 vs 0.66, *P* < 0.001) (Fig. 3). When VER was combined with the established common risk factors, VER improved the C statistic (0.82 vs 0.66, *P* < 0.001), indicating that the combination of VER with common risk factors has a greater potential to predict LR (Fig. 4).

## Discussion

The major findings of this study are as follows; after a single procedure of catheter ablation for paroxysmal or persistent AF, (1) Above half of patients with VER (65.90%) experienced subsequent LR and were associated with lower event-free survival from LR, (2) VER was an independent predictor of LR after adjustment for common risk factors of AF, (3) VER was more sensitive than common risk factors in predicting LR and combination model was superior in predicting LR.

The purpose of catheter ablation is to eliminate underlying cardiac arrhythmia by destroying myocardial tissue through energy. However, due to the complexity of the underlying pathological mechanisms, AF recurs frequently after an initially successful ablation procedure. Reported frequency of LR ranges from 5 to 63%, depending on method and intensity of surveillance, technique used, patient characteristics, and definition of success, with a mean overall successful rate of approximately 70% [18]. In the present study, we found the cumulative LR was about 32.54% at a mean follow-up of 14.71 ± 8.58 months after a single procedure. Among most patients, AF recurred within 7 days.

Recurrence within 3 months following catheter ablation is relatively common regardless of catheter techniques used and is a predictor of LR [5–14]. However, definitions of recurrence time point within the blanking period vary in the reported studies. Arya et al. [9] defined early recurrence as a sustained episode of AF within 7 days immediately after the procedure, while others defined it by a sustained episode of AF within 2 weeks, [5] 1 month, [6, 7] 6 weeks, [8, 10] and 3 months [11–14] during the blanking period. The optimal time to define early recurrence remains to be determined. In this study, we defined sustained AT episode within 7 days as VER since 112 patients (29.63%) experienced it. By using multivariate Cox analysis, VER independently predicted subsequent LR. Mechanisms of arrhythmia recurrence within 3 months of post ablation remain to be fully elucidated and may include reconnection of the PVs, [19] inflammatory response to thermal injury and/or pericarditis, [20, 21] imbalance of the autonomic nervous system, [22, 23] and a delayed effect of AF ablation [23, 24].

The use of 3-month blanking period has been proposed on the assumption that early recurrence will lead to delayed cure and should not prompt immediate re-ablation attempts [15, 25–27]. However, patients with early recurrence and delayed cure were of varied proportion [25–27] and the mechanisms and significance of early arrhythmia remains unclear [19–24]. Given the fact that early recurrence is a strong prognostic factor of LR, delayed re-intervention of tachyarrhythmia within blanking period may be a cause of failure to prevent LR. Indeed, Lellouche et al. [7] evaluated the use of early re-ablation on long-term outcome among patients with early recurrence. After a mean follow-up of 11 ± 11 months, patients with early re-ablation had a lower rate of clinical recurrences. Thus, detection of patients who are at high risk for LR and strategies of aggressive re-intervention may improve at long-term outcome. In our study, VER was more sensitive than common risk factors in prediction of LR. Moreover, when combining VER with common risk factors, it could further improve prediction of LR.

It must be noted that there are limitations in our study. Above all, it is a prospective cohort study and should be validated in large randomized controlled studies. Furthermore, monitoring of atrial tachyarrhythmia recurrence was based on the review of 12-lead electrocardiograms and Holter recordings at follow-up visits. It is likely that more invasive and detailed monitoring of atrial tachyarrhythmia should be offered. Finally, the precise mechanisms of VER and strategies to prevent VER were not investigated and required further research.

## Conclusions

To sum up, the results of this study confirm that VER is observed frequently after a single procedure of catheter ablation and it was strongly associated with LR. Combination between VER and common risk factors could further improve prediction of patients who were at high risk for LR. Whether more aggressively invasive examinations and interventions are helpful for these patients, deserve further studies.

## Abbreviations

AADs: Anti-arrhythmic drugs
AF: Atrial fibrillation
AT: Atrial tachyarrhythmia
LA: Left atria
LR: Long-term recurrence
PVI: Pulmonary vein isolation
PVs: Pulmonary veins
ROC: Receiver operator characteristic
VER: Very early recurrence
